# Mobility of moose—comparing the effects of wolf predation risk, reproductive status, and seasonality

**DOI:** 10.1002/ece3.2598

**Published:** 2016-11-21

**Authors:** Camilla Wikenros, Gyöngyvér Balogh, Håkan Sand, Kerry L. Nicholson, Johan Månsson

**Affiliations:** ^1^ Grimsö Wildlife Research StationDepartment of EcologySwedish University of Agricultural SciencesRiddarhyttanSweden

**Keywords:** carnivore, linearity, movement pattern, predator–prey interaction, speed of movement, ungulate

## Abstract

In a predator–prey system, prey species may adapt to the presence of predators with behavioral changes such as increased vigilance, shifting habitats, or changes in their mobility. In North America, moose (*Alces alces*) have shown behavioral adaptations to presence of predators, but such antipredator behavioral responses have not yet been found in Scandinavian moose in response to the recolonization of wolves (*Canis lupus*). We studied travel speed and direction of movement of GPS‐collared female moose (*n* = 26) in relation to spatiotemporal differences in wolf predation risk, reproductive status, and time of year. Travel speed was highest during the calving (May–July) and postcalving (August–October) seasons and was lower for females with calves than females without calves. Similarly, time of year and reproductive status affected the direction of movement, as more concentrated movement was observed for females with calves at heel, during the calving season. We did not find support for that wolf predation risk was an important factor affecting moose travel speed or direction of movement. Likely causal factors for the weak effect of wolf predation risk on mobility of moose include high moose‐to‐wolf ratio and intensive hunter harvest of the moose population during the past century.

## 1 Introduction

Behavior and movement patterns of animals reflect adaptive responses to environmental conditions as well as inter‐ and intraspecific interactions (Jonsen, Myers, & Flemming, 2003). Consequently, factors such as resource availability, reproductive status, and predation risk affect animal movement (van Beest, Rivrud, Loe, Milner, & Mysterud, 2011; Cederlund, 1989; Cederlund & Sand, 1994; Fortin et al., 2005; Stephens & Peterson, 1984). In predator–prey systems, the risk of predation is an important motivator for certain behavioral decisions. When predators are present, prey species often change behavior to increase their own security (Abramsky, Rosenzweig, & Subach, 2002; Edwards, 1983; Laundré, Hernández, & Altendorf, 2001). For example, prey change habitat selection (Creel, Winnie, Maxwell, Hamlin, & Creel, 2005; Gilliam & Fraser, 2001; Lima & Dill, 1990), group size (Lima, 1995; Winnie & Creel, 2007), and movement behavior (Laundré et al., 2001) as responses to variation in predation risk. However, behavioral responses of prey species may occur only when predators are nearby (Creel & Winnie, 2005).

Prey foraging movements are characterized by frequent turns and shorter steps in the movement path, whereas other activities generally result in more linear movements (Fryxell et al., 2008). Therefore, more concentrated movements can be expected for prey species in habitats with high availability of food versus longer and more directed movements as animals move through areas with low availability of resources (Fryxell et al., 2008) or when fleeing from predators (Wikenros, Sand, Wabakken, Liberg, & Pedersen, 2009). In particular, increased movement rate is likely an advantageous behavior in escaping predators once detected or to minimize the time spent near predators (Gude, Garrott, Borkowski, & King, 2006; Mitchell & Lima, 2002). The presence of predators may also result in lower travel speed as a response to increased vigilance (Berger, 1999; White & Berger, 2001) and thereby suppresses mobility (Lima & Dill, 1990). Such a reduction in movement can also be a beneficial antipredator behavior, because moving animals are generally more easily detected by a predator than are inactive animals (Lima & Dill, 1990). Because these antipredator behaviors are costly, for example, prey species need to trade reduced risk with reduced consumption, theory predicts that prey in systems with low risk or absence of predators should result in a loss of costly antipredator behavior (Blumstein & Daniel, 2005). Likewise, re‐establishment of large predators may result in a resumption of a formerly lost antipredator behavior by prey (Berger, Swenson, & Persson, 2001).

Moose (*Alces alces*) have shown different behavioral adaptations toward presence of wolves (*Canis lupus*) such as adjusting habitat, increased vigilance, and aggressive behavior toward wolves (Berger, 1999; Mech & Peterson, 2003; Stephens & Peterson, 1984; White & Berger, 2001). Wolves preferentially predate on young moose of the year (Sand, Zimmermann, Wabakken, Andrèn, & Pedersen, 2005; Sand et al., 2008). Female moose with calves are therefore expected to be more vigilant and thereby spend less time foraging as compared to lone females and males as a response to wolf presence (Berger, 1999; Berger et al., 2001). However, re‐adaptation to the antipredator behavior of prey may under some circumstances be prevented by anthropogenic influences (Mech, 2012) that are more important for individual survival than predation (Sand, Wikenros, Wabakken, & Liberg, 2006a; Wikenros, Sand, Bergström, Liberg, & Chapron, 2015).

Wolves have been absent from south‐central Scandinavia after extermination for more than 100 years but started to recolonize this region in the 1980s (Wabakken, Sand, Liberg, & Bjärvall, 2001). The population size was estimated to be 289–325 wolves in the winter of 2010/2011 (Wabakken et al., 2011). During the period without wolves, hunter harvest replaced predation as the main mortality source for moose (Lavsund & Sandegren, 1989; Lavsund et al., 2003; Stubsjoen, Saether, Solberg, Helm, & Rolandsen, 2000) likely preventing adaptation of antipredator behavior. For example, moose have not expressed behavioral adjustments to lower wolf hunting success even in territories occupied by wolves for more than 20 years (Gervasi et al., 2013; Sand et al., 2006a; Wikenros et al., 2009). The situation in Scandinavia is therefore interesting because it contrasts with the conditions normally found in protected areas (e.g., national parks) where predation from large predators has not been replaced by hunter harvest. Studies on behaviorally mediated effects on prey by large predators carried out within national parks may not be representative of predator–prey interactions outside national parks (Mech, 2012). Large predators are predicted to have less behaviorally mediated effects on other species in areas where anthropogenic changes have a large impact on several trophic levels (Eriksen et al., 2011; Kuijper et al., 2016; Mech, 2012; Nicholson, Milleret, Månsson, & Sand, 2014; Ripple et al., 2014). Studies in contrasting environments like those found in Scandinavia are therefore important as anthropogenic impact occurs in most parts of the world's wolf range.

We examined the effects of recolonizing wolves on the movement patterns of moose at different spatiotemporal scales. More specifically, we tested whether mobility in terms of travel speed and direction of movement of female moose decreased at a course spatiotemporal scale (annual or seasonal) in order to avoid detection and increased at a finer spatial scale (when wolves are nearby moose) in order to flee from an approaching predator. Ungulate prey are known to adapt to changing environmental conditions affecting resource availability, induced by seasonality as well as reproductive status (Eriksen et al., 2011). Moose calves are the main prey for wolves in Scandinavia year round (Sand et al., 2005, 2008). Therefore, we included time of the year and reproductive status as explanatory variables. We predicted that females with calves would be most likely to change movement patterns in response to wolf predation risk.

## 2 Methods

### 2.1 Study area

The study was conducted in the surroundings of Grimsö Wildlife Research Area (59–60°N, 15–16°E), located in the boreal zone of south‐central Sweden (Rönnegård, Sand, Andrén, Månsson, & Pehrson, 2008) with a study area of approximately 1,000 km^2^. The topography of this rugged plateau is characterized by flat ridges, boulders, and swampy areas with the elevation ranging between 100 and 150 m (digital elevation model, Geographical Data Sweden, GSD, National Land Survey of Sweden). The main land cover type in the area is forest (72%), bogs (18%), lakes and rivers (7%) as well as meadows (3%; Björkhem & Lundmark, 1975). Intensive forest management dominates within the area, with average stand rotation periods of 80–100 years. The main tree species are Scots pine (*Pinus silvestris*), Norway spruce (*Picea abies*), and birches (*Betula pubescens* and *B. pendula*; Månsson, Andrén, Pehrson, & Bergström, 2007). The climate is characterized by continental climate with average temperatures of −5°C in January and 15°C in July (Vedin, 1995). The ground is usually snow‐covered between late November and early April with a mean snow depth of 20 cm in mid‐January (Dahlström, 1995).

During the study period (2007–2010), wolves were continuously present in the study area. The territorial pair (named Uttersberg) established its territory during the winter of 2003/2004 (Wabakken, Aronson, Sand, Strømseth, & Kojola, 2004). Reproduction was confirmed each year from 2004 until 2006 and then again in 2008. During the winter of 2008/2009, a pack of 4–5 wolves was present within the Uttersberg territory, but no reproduction was confirmed in spring 2009. During the following winter (2009/2010), a new territorial pair (named Hedbyn) established in the area, encompassing a major part of the former Uttersberg territory within their territory (Wabakken et al., 2010). Wolf territory sizes reached a maximum of 1,000 km^2^ during the study period (Mattisson et al., 2013). Moose home ranges jointly covered a total area of 410 km^2^ that was partly outside the Uttersberg territory and totally surrounded by the Hedbyn territory. The density of moose was estimated to 1.2 moose/km^2^ in 2002 and 0.8 moose/km^2^ by aerial surveys within the Grimsö Wildlife Research Area (135 km^2^, Rönnegård et al., 2008) and 0.9 moose/km^2^ by pellet count survey within the Uttersberg territory in 2006. The moose population in the area shows high fidelity to the established home ranges and is considered nonmigratory (Cederlund & Okarma, 1988). Other ungulate species are roe deer (*Capreolus capreolus*), with population densities ranging between 1 and 5/km^2^ (Rönnegård et al., 2008), red deer (*Cervus elaphus*), and wild boar (*Sus scrofa*), which occur in low densities (Liberg, Bergström, Kindberg, & Essen, 2010).

### 2.2 GPS locations

Wolves and moose were immobilized by darts from helicopters (see Sand, Wikenros, Wabakken, & Liberg, 2006b; Månsson, Andrén, & Sand, 2011 for details). Handling protocols fulfilled the ethical requirements for research on wild animals in Sweden (decision C281/6 and C315/6). Over the study period, four wolves (the territorial pairs) were collared and the packs were continuously monitored except during a 3‐month period when the Uttersberg pack was replaced by the Hedbyn pack. Female moose were collared in March 2007 (*n* = 20) with an additional 10 females collared in 2010. Wolf GPS collars were programmed for locations with 12‐hr intervals, whereas the GPS collars of the moose took locations every second hour. For this study, we used locations of both species from four consecutive years (2007–2010).

We screened moose GPS data for location errors following the nonmovement method developed by Bjørneraas, Van Moorter, Rolandsen, and Herfindal (2010). We set the distance parameters as ∆ = 100 km and μ = 10 km (three successive locations moving back and forth with high speed limit), and the speed limit was set as ∝ = 1.5 km/hr and turning angle θ = −0.97 (Bjørneraas et al., 2010) to identify spurious locations that formed a spike. We excluded all locations from the 7‐day postcapture to avoid the effect of immobilization on moose behavior (Neumann, Ericsson, Dettki, & Arnemo, 2011). We screened the data using the package Adehabitat (Calenge, 2006) developed for R version 0.95.261 (R Development Core Team, 2012).

### 2.3 Wolf predation risk

Predation risk is the probability of being killed per unit time by the predator (Lima & Dill, 1990), but the mere presence of a predator could be equally important for predicting risk (Hebblewhite & Merrill, 2007). We used three methods to calculate wolf predation risk at different spatiotemporal scales.

First, we calculated an annual predation risk index as the annual home range overlap (%) between all moose home ranges and the wolf territory. We used locations from one of the adult wolves at the time to estimate wolf territory range (*n* = 4) during 2007–2010 (the male in the Uttersberg territory and the female in the Hedbyn territory). This was based on the assumption that the movement and activity of a pair is highly synchronized, with the exception of the pup rearing period (Alfredéen, 2006; Eriksen et al., 2011). We estimated annual territories for wolves and annual moose home ranges using both the 100% minimum convex polygon (MCP; Mohr, 1947) and the 95% fixed kernel (Kernel; Worton, 1987) with the reference technique (href) to calculate the smoothing factor h (Kie et al., 2010). We calculated home ranges using the R library AdehabitatHR (Calenge, 2006). For estimating the area of overlap, the intersect tool and the extension Hawth's Tools in ArcGIS 9.3.1 (ESRI, Redlands, CA, USA) were used. We chose to include both methods for estimates of annual wolf territories and moose home ranges (both annual and seasonal, see below) in order to test for any potential effects of the utilization distribution method on results. The territory sizes (MCP, annual data pooled) were 300–810 and 980 km^2^ for Uttersberg and Hedbyn, respectively.

Second, we calculated a seasonal predation risk index (*S*) according to: *S* = (*w*/(*x*/*y*))/*z* where *w* is the number of wolf locations per individual seasonal moose home range, *x* is the number of wolf locations per season, *y* is the number of potential wolf locations (2 per 24 hr) per season, and *z* is the seasonal moose home range size. Each year was divided into four seasons resulting in a total of 245 individual seasonal moose home ranges (4 years × 4 seasons × number of moose per season) in order to investigate changes in the movement pattern of female moose on a finer temporal scale (Cederlund & Okarma, 1988; Cederlund & Sand, 1994). The seasons used were based on general patterns of moose ecology and behavior: precalving (1 February–30 April), calving (1 May–31 July), postcalving (1 August–31 October), and low activity (1 November–31 January). We excluded from the analysis all individual seasonal moose home ranges that included <95% of the potential moose locations (12 per 24 hr per predefined 3‐month seasonal periods, van Beest et al., 2011).

Third, we calculated instantaneous predation risk as the distance between simultaneous locations (≤1 hr difference) of moose and wolves. Moose locations were taken at 00 and 12 UTC time, and we used travel speed and direction of movement for the following 2‐hr period (until 02 and 14). Wolf locations were taken at 00 and 12 UTC time (for the Uttersberg male; *n* = 17,239) or 23 and 11 (for the Hedbyn female; *n* = 6,407).

### 2.4 Moose mobility

We estimated travel speed (m/hr) and direction of movement (linearity) within all individual seasonal moose home ranges and used these as indices of mobility. We used (1) the straight line distance (m) between consecutive locations and (2) the time elapsed between subsequent location for calculating travel speed (TS) and direction of movement (DM) according to: TS = (*d*_2_ − *d*_1_)/(*t*_2_ − *t*_1_) and DM = *d*_1−3_/(*d*_1−2_ + *d*_2−3_), where *d* is the distance, *t* is the elapsed time, and the subscripts (1, 2, 3) represent consecutive locations. The directional value is always assigned to the second location of each set of three and represents a fraction between 0 and 1. If it is close to 1, it indicates directed movement, whereas values closer to zero indicate movement concentrated within a smaller area (Eriksen et al., 2011). We calculated movement parameters of each study animal with R library “AdehabitatLT” (Calenge, 2006). In order to meet the assumption of normally distributed residuals, travel speed was transformed by ln(*x* + 1) and direction of movement by exp(arcsin(√*x*)) (Eriksen et al., 2011).

### 2.5 Moose reproductive status

Female moose were checked for reproduction in terms of the number of newborn calves at heel in the spring (12 May–4 July), and reproductive individuals were again checked in late summer (26 August–9 September) and at the end of winter the following year (1 April–29 April). We classified females as with or without a calf in each of the four seasons.

### 2.6 Analyses

We conducted all analyses in R version 3.2.2 (R Development Core Team, 2015) using general linear mixed models (GLMM) in the lme4 package (Bates, Maechler, Bolker, & Walker, 2015). The use of GLMM enables modeling of variables measured at multiple time scales with an unbalanced design (which occurred due to collar failure, moose mortality, or exclusion of seasons).

First, we used the average travel speed and average direction of movement of moose as response variables. We used season (precalving, calving, postcalving, and low activity), reproductive status (with calf or without calf), and wolf predation risk as explanatory variables, as well as the interactions between season and reproductive status, season and wolf predation risk, and reproductive status and wolf predation risk. Because the annual index and the seasonal index (continuous variables) both express the potential wolf predation risk, these were entered into the model one at the time, exclusive of each other. The analyses were carried out with MCP estimates than repeated with the kernel estimates.

Second, we used travel speed and direction of movement of moose between consecutive locations (*n* = 23,646) as response variables. We used season, reproductive status, and instantaneous wolf predation risk (continuous variable) as explanatory variables. We also included the interactions between season and reproductive status, season and wolf predation risk, and reproductive status and wolf predation risk. We repeated the analyses using a subset of the data where distances between moose and wolves were __1 km (representing wolves being nearby moose; *n* = 104), 10.5–11.49 km (representing the average distance between wolves and moose; *n* = 1,698), and 20.5–21.49 km (representing wolves being far away from moose: *n* = 272), and the explanatory variable wolf predation risk was used as a three‐level categorical variable.

Moose ID was used as random effect to account for multiple observations of the same individual in all models. We used Akaike information criterion corrected for small sample sizes (AIC_c_) to rank models. Models with ∆AIC_c_ = 0–2 were considered to have equally strong support and models with ∆AIC_c_ = 4–7 to have some support (Burnham & Anderson, 2002).

## 3 Results

The number of individual seasonal moose home ranges used in the analyses of average speed and direction of movements constituted 66% (*n* = 161) of the total number of seasons during the study period (*n* = 245). For 113 seasons, the females had calves and 48 were from females without calves. Seasons were excluded from the analyses due to low number of moose locations (*n* = 58), missing data on wolf exposure data due to collar failure (*n* = 7), or unknown reproductive status (*n* = 19). Due to these exclusions, the total number of female moose used in the analysis was reduced from 30 to 26. The annual predation risk index ranged between 0% and 100% with 58% MCP and 61% kernel seasonal moose home ranges completely inside wolf territories, whereas 14% (MCP) and 12% (kernel) were located outside wolf territories. The seasonal predation risk index ranged between 0 and 1.57 (MCP) and 0–1.70 (kernel).

Data from 205 seasons were used for analyses of travel speed and direction of movement of moose between consecutive locations (*n* = 23,646), excluding data from seasons with unknown reproductive status (*n* = 2,562). The instantaneous predation risk index ranged 119–38,191 m and averaged 10,942 m ± 36 *SE*.

### 3.1 Travel speed

**Figure 1:**
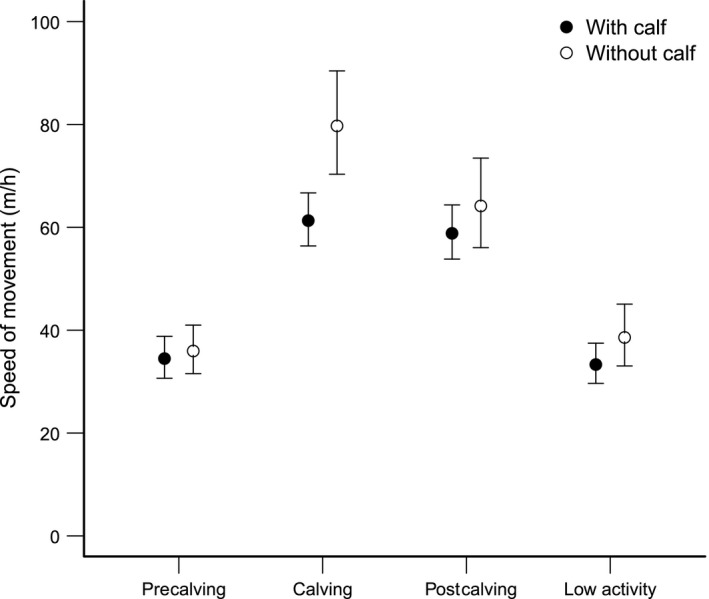
Seasonal variation in travel speed (m/hr, mean ± 95% CI) of female moose (*n* = 26) with or without calves in south‐central Sweden during 2007–2010. Seasons are classified according to precalving (1 February–30 April), calving (1 May–31 July), postcalving (1 August–31 October), and low activity (1 November–31 January)

Travel speed of moose averaged 54.8 m/hr ± 1.5 *SE* during all seasons. The best model explaining variation of travel speed included season and reproductive status as explanatory variables (Table 1). Travel speed was highest during the calving and postcalving seasons and lowest during the low activity season and precalving season (Table 2, Figure 1). Females with calves had a lower travel speed compared to females without calves (Table 2, Figure 1). No support was shown for models including annual wolf predation risk (Table 1). The ΔAIC_c_ for models combining seasonal wolf predation risk with season and reproductive status ranged between 5.1 and 6.2 (Table 1). The models including seasonal wolf predation risk thereby did not provide any net reduction in ΔAIC_c_ (Arnold, 2010) compared to the top models only including season and reproductive status (ΔAIC_c_ = 0, Table 1). The results were independent of the home range estimation method (Table 1). Travel speed between consecutive locations averaged 49.9 m/hr ± 0.6 *SE*, and the best model included the interaction effect between season and reproductive status (Tables 2 and 4). No support was shown for models including the instantaneous wolf predation risk (Table 4). Neither was any support shown for models including the instantaneous wolf predation risk as a categorical variable while using a subset of the data (Table 4).

**Table 1:** General linear mixed models to assess the effect of season (precalving [1 February–30 April], calving [1 May–31 July], postcalving [1 August–31 October] and low activity [1 November–31 January]), reproductive status (with calf or without calf), and either annual wolf predation risk (calculated as the annual home range overlap [%] between all moose home ranges and the wolf territory) or seasonal wolf predation risk (calculated as the number of wolf GPS locations per seasonal moose home range) on moose travel speed (m/hr, *n* = 161) in south‐central Sweden during 2007–2010. Wolf territories and moose home ranges were estimated with both 100% minimum convex polygon and 95% kernel density estimation. For each model, degree of freedom (*df*), difference in AIC_c_ relative to the highest‐ranked model (ΔAIC_c_), and AIC weights (*w*_*i*_) are shown. For simplicity, only models with *w*_*i*_ ≥ 0.001, univariate models, and intercept‐only model are shown

Range method	Model parameters^a^	*df*	ΔAIC_c_	*w*_*i*_
MCP	Season + Reproductive status	7	0	0.850
	Season	6	3.6	0.142
	Season × Reproductive status	10	9.5	0.007
	Intercept only	3	137.6	<0.001
	Reproductive status	4	143.1	<0.001
	Annual wolf predation risk	4	152.1	<0.001
Kernel	Season + Reproductive status	7	0	0.849
	Season	6	3.6	0.142
	Season × Reproductive status	10	9.5	0.007
	Season + Reproductive status + Annual wolf predation risk	8	13.2	0.001
	Intercept only	3	137.6	<0.001
	Reproductive status	4	143.1	<0.001
	Annual wolf predation risk	4	152.1	<0.001
MCP	Season + Reproductive status	7	0	0.786
	Season	6	3.6	0.131
	Season + Reproductive status + Seasonal wolf predation risk	8	5.1	0.061
	Season + Seasonal wolf predation risk	7	7.9	0.015
	Season × Reproductive status	10	9.5	0.007
	Intercept only	3	137.6	<0.001
	Seasonal wolf predation risk	4	142.2	<0.001
	Reproductive status	4	143.1	<0.001
Kernel	Season + Reproductive status	7	0	0.812
	Season	6	3.6	0.136
	Season + Reproductive status + Seasonal wolf predation risk	8	6.2	0.037
	Season + Seasonal wolf predation risk	7	9.2	0.010
	Season × Reproductive status	10	9.5	0.010
	Intercept only	3	137.6	<0.001
	Seasonal wolf predation risk	4	142.4	<0.001
	Reproductive status	4	143.1	<0.001

MCP, minimum convex polygon.

a Moose ID was a random effect to control for multiple observations of the same individual.

**Table 2:** Parameter values for explanatory variables included in the top ranked models (lowest AIC_c_; Tables 1 and 3) for the response variables travel speed (m/hr) and direction of movements of moose in south‐central Sweden during 2007–2010. Seasons were divided according to precalving (1 February–30 April), calving (1 May–31 July), postcalving (1 August–31 October) and low activity (1 November–31 January), and reproductive status as with or without calf

Response variable	Model parameters^a^		β	*SE*	*t*
Travel speed (average per season)		Intercept	3.678	0.058	63.68
	Season	Precalving	−0.010	0.054	−0.19
		Calving	0.623	0.048	12.91
		Postcalving	0.540	0.050	10.88
		Low activity	0		
	Reproductive status	With calf	−0.139	0.043	−3.24
		Without calf	0		
Direction of movement (average per season)		Intercept	3.065	0.028	108.47
	Season	Precalving	−0.008	0.032	−0.24
		Calving	−0.005	0.032	−0.16
		Postcalving	0.009	0.033	0.28
		Low activity	0		
	Reproductive status	With calf	−0.070	0.032	−2.18
		Without calf	0		
	Season × Reproductive status	Precalving:With calf	0.041	0.041	1.01
		Calving:With calf	−0.130	0.039	−3.36
		Postcalving:With calf	0.001	0.039	0.04
		Low activity:Without calf	0		
Travel speed (between locations)		Intercept	2.904	0.050	58.05
	Season	Precalving	−0.249	0.048	−5.22
		Calving	0.805	0.047	17.12
		Postcalving	0.205	0.048	4.23
		Low activity	0		
	Reproductive status	With calf	−0.050	0.047	−1.05
		Without calf	0		
	Season × Reproductive status	Precalving:With calf	0.147	0.058	2.55
		Calving:With calf	−0.216	0.054	−3.98
		Postcalving:With calf	0.085	0.056	1.51
		Low activity:Without calf			
Direction of movement (between locations)		Intercept	3.343	0.037	91.39
	Season	Precalving	0.017	0.039	0.44
		Calving	−0.027	0.038	−0.71
		Postcalving	−0.070	0.039	−1.78
		Low activity	0		
	Reproductive status	With calf	−0.019	0.038	−0.50
		Without calf	0		
	Season × Reproductive status	Precalving:With calf	−0.024	0.047	−0.51
		Calving:With calf	−0.229	0.044	−5.20
		Postcalving:With calf	−0.033	0.045	−0.72
		Low activity:Without calf	0		

a Moose ID was a random effect to control for multiple observations of the same individual.

### 3.2 Direction of movement

**Figure 2:**
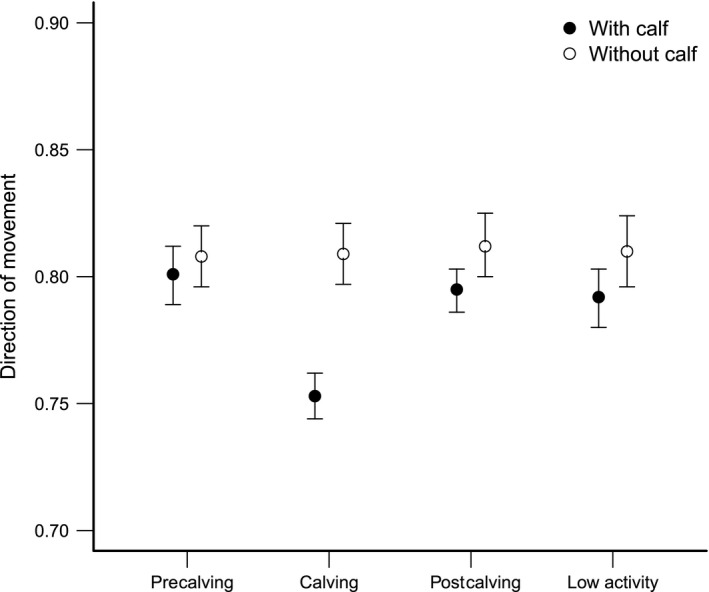
Seasonal variation in direction of movement (mean ± 95% CI) of female moose (*n* = 26) with or without calves in south‐central Sweden during 2007–2010. Seasons are classified according to precalving (1 February–30 April), calving (1 May–31 July), postcalving (1 August–31 October), and low activity (1 November–31 January)

Direction of movement averaged 0.79 ± 0.003 *SE* during all seasons. The best model explaining variation in directions of movement included the interaction effect between season and reproductive status (Table 3). Movements with a low degree of directionality during the calving season were most pronounced for females with calves (Table 2, Figure 2). No support was shown for models including annual or seasonal wolf predation risk (Table 3) irrespective of the home range estimation method used (Table 3). Direction of movement between consecutive locations averaged 0.76 ± 0.002 *SE*, and the best model included the interaction effect between season and reproductive status (Tables 2 and 4). No support was shown for models including the instantaneous wolf predation risk (Table 4). Neither was any support shown for models including the instantaneous wolf predation risk as a categorical variable while using a subset of the data (Table 4).

**Table 3:** General linear mixed models to assess the effect of season (precalving [1 February–30 April], calving [1 May–31 July], postcalving [1 August–31 October] and low activity [1 November–31 January]), reproductive status (with calf or without calf), and either annual wolf predation risk (calculated as the annual home range overlap (%) between all moose home ranges and the wolf territory) or seasonal wolf predation risk (calculated as the number of wolf GPS locations per seasonal moose home range) on the average direction of movement (*n* = 161) of moose in south‐central Sweden during 2007–2010. Wolf territories and moose home ranges were estimated with both 100% minimum convex polygon and 95% kernel density estimation. For each model, degree of freedom (*df*), difference in AIC_c_ relative to the highest‐ranked model (ΔAIC_c_), and AIC weights (*w*_*i*_) are shown. For simplicity, only models with *w*_*i*_ ≥ 0.001, univariate models, and intercept‐only model are shown

Range method	Model parameters^a^	*df*	ΔAIC_c_	*w*_*i*_
MCP	Season × Reproductive status	10	0	0.910
	Season + Reproductive status	7	4.6	0.090
	Season	6	28.6	<0.001
	Reproductive status	4	37.1	<0.001
	Intercept only	3	60.2	<0.001
	Annual wolf predation risk	4	76.3	<0.001
Kernel	Season × Reproductive status	10	0	0.910
	Season + Reproductive status	7	4.6	0.090
	Season	6	28.6	<0.001
	Reproductive status	4	37.1	<0.001
	Intercept only	3	60.2	<0.001
	Annual wolf predation risk	4	75.5	<0.001
MCP	Season × Reproductive status	10	0	0.909
	Season + Reproductive status	7	4.6	0.090
	Season + Reproductive status + Seasonal wolf predation risk	8	13.0	0.001
	Season	7	28.6	<0.001
	Reproductive status	4	37.1	<0.001
	Intercept only	3	60.2	<0.001
	Seasonal wolf predation risk	4	67.4	<0.001
Kernel	Season × Reproductive status	10	0	0.909
	Season + Reproductive status	7	4.6	0.090
	Season + Reproductive status + Seasonal wolf predation risk	8	12.7	0.002
	Season	6	28.6	<0.001
	Reproductive status	4	37.1	<0.001
	Intercept only	3	60.2	<0.001
	Seasonal wolf predation risk	4	67.5	<0.001

MCP, minimum convex polygon.

a Moose ID was a random effect to control for multiple observations of the same individual.

**Table 4:** General linear mixed models to assess the effect of season (precalving [1 February–30 April], calving [1 May–31 July], postcalving [1 August–31 October] and low activity [1 November–31 January]), reproductive status (with calf or without calf) and instantaneous wolf predation risk (calculated as distance between moose and wolves at simultaneous GPS locations of collared individuals) on moose travel speed (m/hr) and direction of movement in south‐central Sweden during 2007–2010. Analyses was conducted using all available data (*n* = 23,646) and a subset of the data (*n* = 1,804) where distances between moose and wolves __1, 10.5–11.49, and 20.5–21.49 km were included. For each model, degree of freedom (*df*), difference in AIC_c_ relative to the highest‐ranked model (ΔAIC_c_), and AIC weights (*w*_*i*_) are shown. For simplicity, only models with *w*_*i*_ ≥ 0.001, univariate models, and intercept‐only model are shown

Data	Response variable	Model parameters^a^	*df*	ΔAIC_c_	*w*_*i*_
All	Travel speed	Season × Reproductive status	10	0	1.000
		Season	6	56.6	<0.001
		Reproductive status	4	1,557.9	<0.001
		Intercept only	3	1,558.7	<0.001
		Instantaneous wolf predation risk	4	1,578.8	<0.001
All	Direction of movement	Season × Reproductive status	10	0	1.000
		Season	6	59.4	<0.001
		Reproductive status	4	169.6	<0.001
		Intercept only	3	218.5	<0.001
		Instantaneous wolf predation risk	4	243.6	<0.001
Subset	Travel speed	Season	6	0	0.656
		Season × Reproductive status	10	2.3	0.208
		Season + Reproductive status	7	3.3	0.126
		Season + Instantaneous wolf predation risk	8.7	8.7	0.009
		Season + Reproductive status + Instantaneous wolf predation risk	11.9	11.9	0.002
		Intercept only	3	138.4	<0.001
		Reproductive status	4	143.3	<0.001
		Instantaneous wolf predation risk	4	148.0	<0.001
Subset	Direction of movement	Season × Reproductive status	10	0	0.497
		Reproductive status	4	2.6	0.134
		Season	6	2.7	0.131
		Intercept only	3	2.9	0.119
		Season + Reproductive status	7	3.1	0.107
		Reproductive status + Instantaneous wolf predation risk	6	10.2	0.003
		Instantaneous wolf predation risk	5	10.3	0.003
		Season + Instantaneous wolf predation risk	8	10.4	0.003
		Season + Reproductive status + Instantaneous wolf predation risk	9	11.1	0.002

a Moose ID was a random effect to control for multiple observations of the same individual.

## 4 Discussion

This study gave no support for the hypothesis that the re‐establishment of wolves in Sweden has affected mobility in terms of either travel speed or direction of movement of female moose. However, both travel speed and direction of movement were affected by seasonal changes and reproductive status. Travel speed of females was highest during the calving season and the postcalving season, and reduced during the rest of the year. This is in line with previous studies that show that moose movement rates peak sometime during May to September (Cederlund, 1989; Eriksen et al., 2011; Vander Wal & Rodgers, 2009) and gradually reduce from October through November (Eriksen et al., 2011) to the lowest around February (Cederlund, 1989). This variation in movement rates follows seasonal changes because activity patterns are highly correlated with food quality and availability (Cederlund, 1989; Cederlund, Bergström, & Sandegren, 1989; Renecker & Hudson, 1986). Also, female moose accompanied by calves moved less directionally than lone females and this difference occurred mainly during the calving season. Eriksen et al. (2011) also observed minimal variation in the directionally of movements of female moose between seasons, except for a reduction in June, which they explained were restricted movements due to the limited movement abilities of newborn calves. We found no support for the prediction that females with calves should be more prone to changes in their mobility in relation to wolf predation risk than females without calves.

Although behaviorally mediated effects have been examined for several traits in Scandinavian moose as a response to wolf recolonization, no significant effect on moose behavior has so far been shown. Behavioral effects investigated include moose defense behavior against attacking wolves (Gervasi et al., 2013; Sand et al., 2006a, 2006b; Wikenros et al., 2009), daily and seasonal activity patterns between wolves and moose (Eriksen et al., 2008, 2011), and moose habitat selection (Nicholson et al., 2014). The results from our present study add further support for the view that recolonization of a predator to previously inhabited areas does not always and universally lead to changes in the behavior of their prey species. In our study area, wolves first established a territory 3 years prior to the GPS collaring of moose and the data collected span over a 3‐ to 6‐year period after the establishment of wolves. An alternative interpretation of our results is therefore that the time of exposure of wolves has been too short to initialize a behavioral response in the moose population. However, there are a number of studies that have reported rapid behavioral responses of prey as a result of resumed levels of predation risk (Berger, 1999; Berger et al., 2001; Hunter & Skinner, 1998; Laundré et al., 2001), suggesting that behaviorally mediated effects may show a rapid manifestation in the prey population.

Our results contrast with results from a number of studies in North America, showing that prey species, for example, elk (*Cervus elaphus*), bison (*Bison bison*), and moose, can show behavioral adaptation toward the presence of wolves with adjusting habitat (Stephens & Peterson, 1984), increased vigilance (Berger, 1999; Berger et al., 2001; White & Berger, 2001), shift in feeding and birthing sites (Edwards, 1983, Berger et al., 2001), or aggressive behavior (Mech & Peterson, 2003). In this perspective, our results may be considered as rather surprising. However, not all studies investigating behaviorally mediated effects on prey as a result of resumed predation risk by wolves have been able to confirm that these effects always exist even in North America (Kauffman, Brodie, & Jules, 2010). We suggest that there are several causal factors that together may explain why we do not find support for the hypothesis that the recolonization of wolves will lead to behaviorally mediated effects on prey in Scandinavia. The moose population in Scandinavia has experienced a strong decline during the 18th to early 20th century (Niedziałkowska et al., 2015), which to some extent was contemporary to the extinction of wolves from central Scandinavia in the mid‐19th century. As a result, the rebounding of the moose population starting in the late 19th century became mainly regulated by hunter harvest and has since then been by far the most important mortality factor of the moose population (Lavsund et al., 2003). Although wolves have been present in some areas in Scandinavia for more than 20 years (Gervasi et al., 2013; Sand et al., 2006a) and have been shown to prey mainly on moose with relatively high kill rates (Sand et al., 2005, 2008; Zimmermann, Sand, Wabakken, Liberg, & Andreassen, 2015), hunter harvest typically remains the main mortality factor even within most Scandinavian wolf territories (Wikenros et al., 2015). Also, the relatively large current size of wolf territories (Mattisson et al., 2013) and high density of moose (Sand et al., 2012) both contribute to create low ratios of wolf‐to‐moose (Eriksen et al., 2011; Sand et al., 2012) in Scandinavia. A direct consequence of the low wolf‐to‐moose ratio is that the frequency of encounters between wolves and any particular moose individual is low (Eriksen et al., 2008) that is also confirmed in this study where the distance between moose and wolves on average was 11 km.

In contrast to the predator–prey system in Scandinavia, most of the studies in North America have been carried out in protected areas such as national parks (Mech, 2012). Therefore, one factor that may explain these variable results may be the degree of anthropogenic impact on the ecosystem and on prey populations in particular. The impact of hunter harvest as an evolutionary force relative to predation by large predators has so far received little attention but is limited mainly by the access to empirical long‐term data on morphology and behavior (Darimont et al., 2009; Fenberg & Roy, 2008). Further, because most of the prey populations now exposed to recolonizing populations of large predators (Chapron et al., 2014) is, and for a long time has been, under strong anthropogenic influence, the results received from the current and previous studies in Scandinavia on behaviorally mediated effects on prey may be more the norm as compared to studies carried out in protected areas.

We conclude that in a moose population where hunter harvest is the main mortality factor, the movement pattern of female moose was mainly influenced by external factors and reproductive status, and not by the return of their long absent natural predator, the wolf.

## Conflict of Interest

None declared.
